# Association between rest-activity rhythm and diabetic retinopathy among US middle-age and older diabetic adults

**DOI:** 10.3389/fendo.2024.1440223

**Published:** 2024-09-16

**Authors:** Zhijie Wang, Mengai Wu, Haidong Li, Bin Zheng

**Affiliations:** ^1^ Department of Retina Center, Eye Hospital and School of Ophthalmology and Optometry, Wenzhou Medical University, Hangzhou, China; ^2^ National Clinical Research Center for Ocular Diseases, Eye Hospital, Wenzhou Medical University, Wenzhou, China

**Keywords:** diabetic retinopathy, rest-activity rhythm, circadian rhythm, fragmented rhythm, NHANES

## Abstract

**Background:**

The disruption of circadian rhythm has been reported to aggravate the progression of diabetic retinopathy (DR). Rest-activity rhythm (RAR) is a widely used method for measuring individual circadian time influencing behavior. In this study, we sought to explore the potential association between RAR and the risk of DR.

**Methods:**

Diabetic participants aged over 40 from 2011-2014 National Health and Nutrition Examination Survey (NHANES) were enrolled. Data from the wearable device ActiGraph GT3X was used to generate RAR metrics, including interdaily stability (IS), intradaily variability (IV), most active 10-hour period (M10), least active 5-hour period (L5), and Relative amplitude (RA). Weighted multivariable logistic regression analysis and restricted cubic spline analysis were conducted to examine the association between RAR metrics and DR risk. Sensitivity analysis was also conducted to examine the robustness of the findings. An unsupervised K-means clustering analysis was conducted to identify patterns in IV and M10.

**Results:**

A total of 1,096 diabetic participants were enrolled, with a DR prevalence of 20.53%. The mean age of participants was 62.3 years, with 49.57% being male. After adjusting covariates, IV was positively associated with DR (β: 3.527, 95%CI: 1.371-9.073). Compared with the lowest quintile of IV, the highest quintile of IV had 136% higher odds of DR. In contrast, M10 was negatively associated with DR (β: 0.902, 95%CI: 0.828-0.982), with participants in the highest M10 quintile showing 48.8% lower odds of DR. Restricted cubic spline analysis confirmed that these associations were linear. Meanwhile, sensitivity analysis confirmed the robustness. K-means clustering identified three distinct clusters, with participants in Cluster C (high-IV, low-M10) had a significantly higher risk of DR comparing with Cluster A (low-IV, high-M10).

**Conclusion:**

A more fragmented rhythm and lower peak activity level might be associated with an increased risk of DR. These findings indicate that maintaining a more rhythmic sleep-activity behavior might mitigate the development of DR. Further research is necessary to establish causality and understand the underlying mechanisms, and focus on whether interventions designed to enhance daily rhythm stability and increase diurnal activity level can effectively mitigate the risk of progression of DR.

## 1 Introduction

Diabetic retinopathy (DR), is the leading cause of vision impairment and blindness among work-age adults, characterized by the progressive retina vascular damage as a severe complication of diabetes mellitus. Paralleling the rise of diabetes, the prevalence of DR had been increasing globally. According to a recent meta-analysis, the global prevalence of DR among the diabetic population is 22.27%, and the number of DR is projected to reach 160.50 million by 2045 (1). DR manifests through series of stages, from microaneurysms abnormalities to proliferative DR marked by neovascularization and retinal detachment (2). Understanding the risk factors and pathophysiological mechanisms of DR is crucial for developing effective prevention and treatment strategies.

Circadian rhythm is an intrinsic 24-hour cycle regulating various biological processes in organisms. One critical output of circadian rhythm is the rest-activity rhythm (RAR), which encompasses the patterns of physical activity and rest throughout the day and night (2). Disturbance of RAR, often resulting from irregular sleep patterns, shift work, or exposure to artificial light (3–5), has been implicated in various metabolic diseases and cardiovascular disorders, such as hypertension, diabetes, and so on (2, 6, 7).

Emerging evidence indicates that the disruption of circadian rhythm may also influence the diverse facets of DR, from alterations in clock genes to changes in rhythmic clinical manifestations (8, 9). Notably, a remarkable decrease of a diverse array of clock genes were observed in retina of diabetes rodent model (10, 11). Our recent study revealed that diabetes reshapes the rhythmic profile of retinal transcriptome (12). Aberrant expression or loss of core clock genes could accelerate retinal endothelial dysfunction and increase retinal permeability (13). Furthermore, disturbed rhythms in retinal structures and physiological functions, such as retinal thickness and melatonin rhythm, have been observed in DR (14). A recent cross-sectional study explored alteration of circadian rhythm in DR by examining 24-hour melatonin secretion, intrinsically photosensitive retinal ganglion cell (ipRGC) and RAR (15). The results indicated that DR patients exhibited lower melatonin output and reduced ipRGC function. However, due to the small sample size (n=25), no significant change in RAR was observed. In the present study, we aimed to explore the association between RAR metrics and DR based on a large, population-based cohort, hypothesizing that irregular RAR may be a potential risk factor for the development and progression of DR. This will be investigated through a cross-sectional study among middle-age or older diabetic participants (age≥40) within the U.S. National Health and Nutrition Examination Survey (NHANES).

## 2 Methods

### 2.1 Study population

NHANES is a complex, multi-staged national survey in the United States, that investigates the health and nutrition status of U.S. civilian population through personal interviews, physical examinations and laboratory tests (16). This survey is conducted in two-year cycles. The NHANES protocols were approved by the NCHS Research Ethics Review Board (Protocol #2011-17), and written consent was received from each participant.

**Figure 1:**
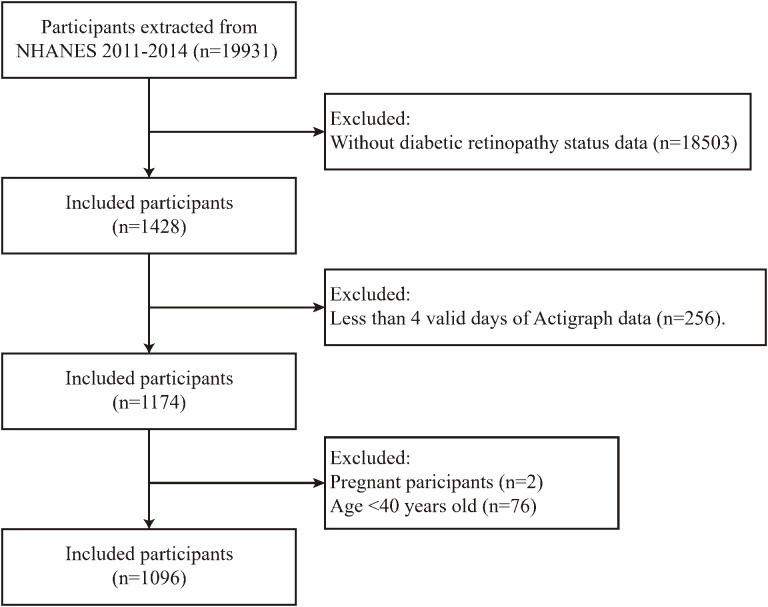
Flow chart of the sample screening process.

For present study, we limited the participants to those from 2011-2014 cycles of NHNAES. Participants with the following characteristics were excluded: a) absence of diabetes mellitus; b) age <40 years old; c) those with fewer than 4 days of valid accelerometer data; d) pregnancy. As shown in **Figure 1**, a total of 1096 participants met inclusion criteria for the subsequent analysis.

### 2.2 RAR metrics

According to NHANES protocol, all participants aged 6 years and older were required to wear the physical activity monitor (PAM) ActiGraph GT3X+ accelerometer (Pensacola, FL) for seven consecutive days to collect objective data. The accelerometer data, recorded at a frequency of 80 Hz, and ambient light measurements, taken at 1 Hz, were aggregated on a per-minute basis for each participant, specified in Monitor-Independent Movement Summary (MIMS) units.

The first and last days of accelerometer data were omitted due to the incomplete 24-h periods. According to the previous research, the raw data were pre-processed using accelmissing R package (17). A valid day was defined as having more than 16 hours of wearing time, and subjects were required to have at least four valid days of data to be included in the analysis. RAR metrics are reliable variables for quantifying the strength, timing, and regularity of sleep and physical activity patterns, partially reflecting the objective behavioral manifestation of daily rhythm in response to environmental cues (18). In this study, we employed non-parametric methods to generate RAR metrics using nparACT R package (19). The key RAR metrics included:1) Interdaily Stability (IS), which assesses the consistency of the rest-activity pattern with the 24h light-dark cycle; 2) intradaily variability (IV), which measures the fragmentation of the 24-hour rhythm, with higher IV values indicating greater rhythm fragmentation, commonly associated with frequent snapping or inefficient sleep; 3) Most active 10-hour period (M10), which measures the average activity of ten consecutive hours with the peak activity among 24-hour period; 4) Least active 5-hour period (L5), measures the average activity of five consecutive hour with nadir activity; 5) Relative amplitude(RA), which demonstrates the relative difference between M10 and L5, with higher RA values indicating greater circadian rhythm strength.

### 2.3 Definition of diabetes mellitus and diabetic retinopathy

Diabetes mellitus is defined according to the following criteria: 1) glycohemoglobin HbA1c ≥ 6.5%; 2) fasting glucose ≥ 7.0mmol/L; 3) self-reported previous diagnosis of diabetes by a doctor. Participants were classified as having DR if they answered ‘yes’ to: ‘Has a doctor ever informed you that your eyes are affected by diabetes or that you have retinopathy?’

### 2.4 Covariates

The following covariates were included: age (40-60, >60 years), sex (male, female), marital status (never married, married or living with a partner, widowed/divorced/separated), education level (<high school, high school or equivalent, >high school), poverty income ratio (PIR, <1.3,1.3-3.5,>3.5) race ethnicity (non-Hispanic white, other), self-reported sleep problems (yes, no), hypertension (yes, no), and hyperlipidemia (yes, no). Missing values for covariates were imputed with multiple imputation by chained equations with the ‘mice’ R package. We generated five imputed datasets with the random forest imputation algorithm and subsequently pooled the results following Rubin’s standard rules.

### 2.5 Statistical analysis

In the present study, all statistical analyses were conducted with consideration of the survey weights based on the NHANES protocol. Participant characteristics were demonstrated using descriptive statistical analyses based on the presence of DR. Continuous variables were presented as weighted mean ± SD, and differences between groups were compared using one-way ANOVA. Categorical variables were presented as weighted percentage, and differences were compared using the Rao-Scott chi-square test. To investigate the association between the risk of DR and each RAR metric, weighted logistic regression analysis was conducted. RAR metrics were categorized into quintiles, and the first quintile was set as the reference. Three regression models were generated: Model 1 was the crude model without any adjustments. Model 2 was adjusted for the age, sex, race, marital status, PIR. Model 3 was further adjusted for hypertension, hyperlipidemia, and sleep problems. In the fully adjusted model, the non-linear relationship was explored using the restricted cubic spline (RCS) method with four knots, setting RAR metrics as the exposure and the risk of DR as the outcome. To identify patterns in IV and M10, an unsupervised K-means clustering analysis was conducted with R.

For subgroup analysis, participants were stratified by age (40-60, >60), sex (male, female), education level (<high school, high school or equivalent, >high school), and race ethnicity (non-Hispanic white, other) in the fully adjusted model. The interaction between RAR metrics and potential modifiers were calculated to examine the differences in effect across subgroups (p for interaction). To assess the robustness of the main findings, sensitivity analysis was performed after excluding the participant with any missing covariates.

All statistical analyses were performed using R (version 4.2.2), and two-side p-value <0.05 was considered statistically significant.

## 3 Results

### 3.1 Baseline characteristic

In this study, a total of 1,096 eligible participants with diabetic mellitus were included (**Figure 1**), representing an estimated weighted population of 16,930,542 in the United States. The mean age of participants was 62.3 years, with 49.57% being male. Detailed characteristics of the participants are summarized in **Table 1**, covering a variety of social-culture backgrounds in the United States. The prevalence of DR among the valid participants was 20.53%. Notably, the majority of participants were non-Hispanic white (63.27%), and more than (76.16%) had a high school degree or above. In terms of marital status, the percent of married or live with a partner is 60.35% in this cohort. The rest-activity rhythm was quantified into five parameters: IS, IV, RA, L5 and M10. Compare to participants without DR, those with DR had a lower M10 level (p=0.035) and tended to have higher IV level, though this difference was not statistically significant (p=0.065). Additionally, participants with DR were more likely to be never married and had a lower prevalence of hypertension (p=0.044 and 0.048, respectively).

**Table 1:** Baseline characteristics of participants in NHANES 2011-2014 (N=1096).

Characteristic	total(N=1096)	Without DR (N=871)	With DR (N=225)	p
Age, n (%)				0.784
40-60	373(40.49)	301(40.24)	72(41.72)	
≥60	723(59.51)	570(59.76)	153(58.28)	
Sex, n (%)				0.185
Female	548(50.43)	452(51.55)	96(44.92)	
Male	548(49.57)	419(48.45)	129(55.08)	
Race, n (%)				0.115
Non- Hispanic White	387(63.27)	314(64.44)	73(57.52)	
other	709(36.73)	557(35.56)	152(42.48)	
Education level, n (%)				0.074
< High school	370(23.83)	290(22.89)	80(28.49)	
High School Grad/ GED or Equivalent	256(25.51)	199(24.53)	57(30.35)	
> High school	470(50.65)	382(52.57)	88(41.17)	
Marital status, n (%)				0.044*
Married or living with a partner	598(60.35)	485(61.43)	113(55.04)	
Never married	96(7.96)	77(6.92)	19(13.14)	
Widowed, divorced, separated	402(31.69)	309(31.66)	93(31.82)	
PIR, n (%)				0.126
<1.3	447(28.70)	347(27.08)	100(36.66)	
1.3-3.5	415(39.69)	332(40.14)	83(37.50)	
>3.5	234(31.61)	192(32.78)	42(25.84)	
Smoke, n (%)				0.932
never	550(48.89)	444(48.63)	106(50.18)	
former	393(37.23)	304(37.51)	89(35.84)	
now	153(13.88)	123(13.86)	30(13.97)	
Hypertension, n (%)				0.048*
Yes	257(23.39)	216(24.58)	41(17.54)	
No	839(76.61)	655(75.42)	184(82.46)	
Hyperlipidemia, n (%)				0.938
No	138(9.877)	112(9.901)	26(9.758)	
Yes	958(90.123)	759(90.099)	199(90.242)	
Sleep problem, n (%)				0.423
No	692(59.14)	553(58.51)	139(62.26)	
Yes	404(40.86)	318(41.49)	86(37.74)	
IS, mean ± SD	0.37 ± 0.00	0.37 ± 0.00	0.36 ± 0.01	0.163
IV, mean ± SD	0.64 ± 0.01	0.64 ± 0.01	0.68 ± 0.02	0.065
RA, mean ± SD	0.79 ± 0.01	0.80 ± 0.01	0.78 ± 0.01	0.154
L5, mean ± SD	1.08 ± 0.04	1.09 ± 0.04	1.08 ± 0.06	0.939
M10, mean ± SD	9.64 ± 0.14	9.76 ± 0.14	9.06 ± 0.32	0.035*

PIR, poverty income ratio. IS, interdaily stability. IV, intradaily variability. M10, most active 10-hour period. L5, least active 5-hour period. RA, relative amplitude. *p value<0.05.

### 3.2 Association between RAR metrics and DR

**Figure 2:**
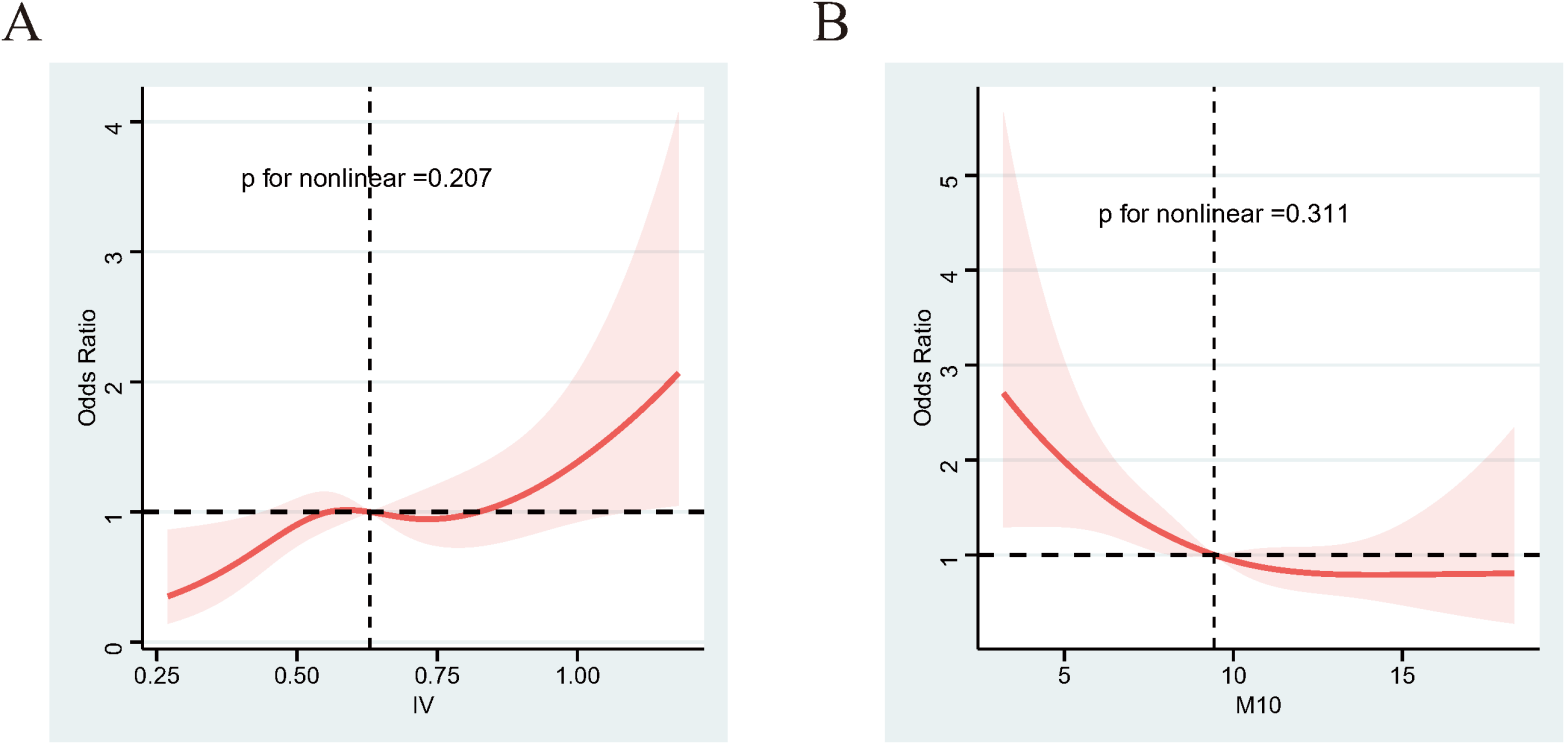
RCS results between IV/M10 and DR. **(A)** IV and the risk of DR. **(B)** M10 and the risk of DR. Adjusted for age, sex, race, education level, marital status, PIR, sleep problem, and hyperlipidemia and hypertension. The shaded part represents the 95%CI. RCS, restricted cubic spline.

To investigate the association between RAR and DR, we conducted weighted multivariable logistic regression analyses on the RAR metrics. As shown in **Table 2**, after adjusting for a comprehensive set of confounders, the results of Model 3 demonstrated a positive association between IV and the risk of DR (OR=3.527; 95% CI, 1.371-9.073; p=0.011). Additionally, the value of M10 was negatively associated with DR when adjusted for sociodemographic factors and comorbidity (**Table 2**, Model 3, OR=0.902; 95% CI, 0.828-0.982; p=0.02). Consistently, when we categorized RAR metrics by dividing the values into quintiles, the participants in the fifth quintile (Q5) of IV were 136% more likely to develop DR than those in the first quintile (Q1) (**Table 3**, Model 3; OR=2.360; p for trend = 0.047). The risk of DR in the Q5 group of M10 showed significant reductions compared to the Q1 group, with a decrease of 49% (**Table 3**, Model 3; OR=0.512; p for trend = 0.024). To explore the nonlinear relationship between IV/M10 and the risk of DR, we conducted RCS analysis. No nonlinear association was detected for either IV or M10 (**Figure 2**, p for nonlinearity = 0.207 and 0.311, respectively). However, as demonstrated in **Figure 2**, these two curves also do not completely fit a linear relationship. For association between IV and the risk of DR, the curve is relatively stable over most of the range but shows a significant upward trend at high IV values (IV>0.75). For association between IV and the risk of DR, when M10 <9, the OR value decrease significantly as M10 increases, whereas when M10 value exceed 9, this downward trend gradually stabilizes. In summary, as IV value increases and M10 value decreases, the risk of developing DR tends to rise.

**Table 2:** Association between RAR metrics and DR (N=1096).

24-h Rest-activity rhythm variables	Model 1		Model 2		Model 3	
	OR (95%CI)	p	OR (95%CI)	p	OR (95%CI)	p
RA	0.327(0.074,1.443)	0.135	0.375(0.087,1.610)	0.178	0.366(0.084,1.589)	0.170
IS	0.249(0.035,1.786)	0.160	0.292(0.047,1.812)	0.177	0.281(0.044,1.794)	0.170
IV	2.971(1.025,8.613)	0.045*	3.554(1.373,9.202)	0.011*	3.527(1.371,9.073)	0.011*
L5	0.993(0.813,1.211)	0.940	0.969(0.787,1.194)	0.758	0.973(0.789,1.199)	0.787
M10	0.916(0.839,1.001)	0.052	0.903(0.830,0.982)	0.020*	0.902(0.828,0.982)	0.020*

Model 1: crude model, without any adjustment.

Model 2: adjusted for age, sex, education level, marital status, PIR.

Model 3: adjusted for age, sex, education level, marital status, PIR, sleep problem, and hyperlipidemia and hypertension.

OR, odds ratios. CI, confidence interval. RAR, rest-activity rhythm. RA, relative amplitude. IS, interdaily stability. IV, intradaily variability. M10, most active 10-hour period. L5, least active 5-hour period.

*p value<0.05.

**Table 3:** Association between quintiles of RAR metrics and DR.

24-h Rest-activity rhythm variables	Model 1 OR (95% CI)	Model 2 OR (95% CI)	Model 3 OR (95% CI)
IS			
Q1	ref	ref	ref
Q2	0.957(0.512,1.790)	1.022(0.538,1.942)	1.006(0.530,1.909)
Q3	1.328(0.664,2.656)	1.515(0.751,3.056)	1.511(0.748,3.049)
Q4	0.435(0.236,0.800)	0.470(0.264,0.838)	0.466(0.258,0.842)
Q5	0.895(0.480,1.666)	0.938(0.538,1.638)	0.924(0.521,1.639)
p for trend	0.211	0.225	0.222
IV			
Q1	ref	ref	ref
Q2	1.471(0.804,2.693)	1.737(0.943,3.198)	1.737(0.933,3.232)
Q3	0.961(0.501,1.843)	1.150(0.571,2.315)	1.157(0.575,2.331)
Q4	1.185(0.489,2.869)	1.438(0.578,3.581)	1.443(0.578,3.599)
Q5	1.949(1.016,3.738)	2.368(1.214,4.621)	2.360(1.205,4.621)
p for trend	0.132	0.047*	0.047*
M10			
Q1	ref	ref	ref
Q2	0.686(0.317,1.483)	0.696(0.327,1.481)	0.696(0.325,1.488)
Q3	0.416(0.193,0.896)	0.449(0.213,0.948)	0.444(0.207,0.951)
Q4	0.369(0.177,0.772)	0.343(0.161,0.729)	0.343(0.161,0.728)
Q5	0.593(0.248,1.419)	0.518(0.212,1.263)	0.512(0.208,1.260)
p for trend	0.063	0.026*	0.024*
L5			
Q1	ref	ref	ref
Q2	1.247(0.587,2.652)	1.233(0.591,2.574)	1.233(0.584,2.603)
Q3	1.134(0.496,2.593)	1.083(0.503,2.333)	1.072(0.495,2.319)
Q4	1.720(0.839,3.526)	1.715(0.856,3.436)	1.730(0.848,3.531)
Q5	1.190(0.642,2.205)	1.102(0.600,2.022)	1.113(0.601,2.059)
p for trend	0.386	0.544	0.515
RA			
Q1	ref	ref	ref
Q2	0.885(0.450,1.741)	0.905(0.450,1.821)	0.905(0.445,1.840)
Q3	0.740(0.420,1.305)	0.769(0.442,1.336)	0.762(0.438,1.325)
Q4	0.599(0.317,1.133)	0.637(0.331,1.226)	0.632(0.328,1.220)
Q5	0.595(0.265,1.336)	0.608(0.271,1.365)	0.602(0.266,1.363)
p for trend	0.09	0.111	0.108

Model 1: crude model, without any adjustment.

Model 2: adjusted for age, sex, education level, marital status, PIR.

Model 3: adjusted for age, sex, education level, marital status, PIR, sleep problem, and hyperlipidemia and hypertension.

OR, odds ratios. CI, confidence interval. RAR, rest-activity rhythm. RA, relative amplitude. IS, interdaily stability. IV, intradaily variability. M10, most active 10-hour period. L5, least active 5-hour period. Ref, reference. *p value<0.05.

### 3.3 Stratified and sensitivity analyses

To explore the impact of age, sex, race and education level on the findings, we performed the subgroup analysis based on different features (**Supplementary Tables 1**, **2**). The IV quintiles displayed interactions with different education level subgroups (p for interaction = 0.014). The negative association between IV and DR was predominantly significant among the population with a high school education level or below. 

In sensitivity analyses, participants with any missing covariates were excluded, and the analysis was re-performed on the remaining 938 participants. The association between IV and M10 and the risk of DR persisted, confirming that the main findings were reliable (**Supplementary Table 3**).

### 3.4 Unsupervised cluster analysis of IV and M10 in DR

**Figure 3:**
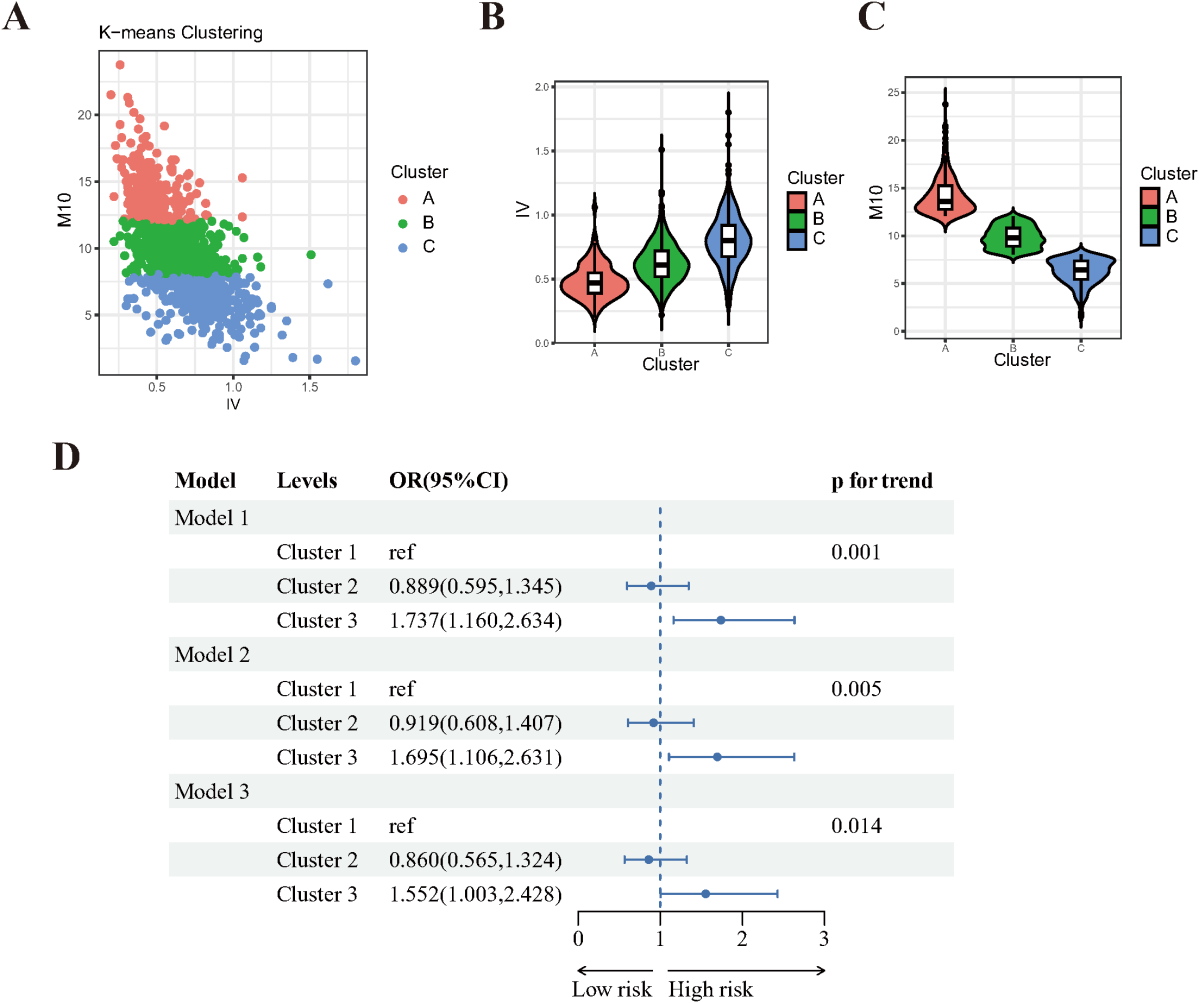
Unsupervised Cluster analysis results of IV and M10 in DR. **(A)** three distinct clusters were identified by K-means clustering. **(B)** violin plot of IV distribution among clusters. **(C)** violin plot of IV distribution among clusters. **(D)** Forest plot of clusters and the risk of DR. Model 1: crude model, without any adjustment. Model 2: adjusted for age, sex, education level, marital status, PIR. Model 3: adjusted for age, sex, education level, marital status, PIR, sleep problem, and hyperlipidemia and hypertension.

Subsequently, we performed an unsupervised K-means clustering analysis to cluster 1,096 participants based on their IV and M10 values. As depicted in **Figure 3A**, three distinct clusters were identified: Cluster A (n=230), Cluster B (n=519) and Cluster C (n=347). These clusters demonstrated significant differences in IV and M10 distribution, with Cluster A exhibiting lower IV and higher M10, while Cluster C had higher IV and lower M10 values (**Figures 3B, C**).

The baseline characteristics of participants in these three clusters are summarized in **Supplementary Table 4**. There were significant differences in age, sex, race, education level, PIR and DR risk among the clusters. Notably, participants in Cluster C (high-IV, low-M10) are more prone to have DR.

Logistic regression analyses were conducted to evaluate the association between these clusters and the risk of DR. After adjusting for a comprehensive set of covariates, participants in Cluster C (high-IV, low-M10) were found to be 55% more likely to have DR compared to those in Cluster A (low-IV, high-M10)(p for trend=0.014, **Figure 3D**). This result further supports the above finding that the risk of DR increases as M10 decreases and IV increases.

## 4 Discussion

The present study uncovered the association between DR and RAR parameters in a representative U.S. working-age or older diabetes population. Notably, our findings demonstrated that the risk of DR increases with the higher IV value and lower M10 value. This indicates that fragmented rhythms and lower peak activity are more common among DR participants. These findings highlight the potential advantages of maintaining a regular sleep-activity rhythm and having high activity levels during the daytime in terms of delaying the onset of DR.

It is well documented that circadian rhythm plays a crucial role in regulating glucose homeostasis (20). Previous studies have revealed that the disruptions of circadian rhythm, induced by lifestyle factors such as shift work (21) and social jetlag (22), are associated with type 2 diabetes. Glucose homeostasis is essential for maintaining the normal physiological function of retinal cells. Hyperglycemia triggers a cascade of complex pathophysiological changes in retinal cells (23). For example, chronic hyperglycemia can cause endothelial dysfunction, pericyte loss, and the perturbations of endothelial glycocalyx in the retina (24, 25). In addition, hyperglycemia activates retinal microglia and Müller cell, inducing the release of various inflammatory cytokines and vascular growth factors (26, 27). The production of reactive oxygen species and the formation of advanced glycation end products also increase under poorly controlled blood glucose. These processes exacerbate retinal vascular damage, promote neovascularization, and ultimately lead to the progression of DR (28).

Additionally, clinical findings and laboratory studies have indicated that circadian rhythm disturbances are directly associated with DR (29). Melatonin, a hormone acting as a dark signal for the retina, was found to be decreased in DR patients (15, 30). Furthermore, DR patients suffer from ipRGC dysfunction (15), which is a crucial component of the light entrainment in synchronization of the central clock to environment cues (31). Our previous study found that circadian oscillations of the transcriptomic profile in the retina were reshaped in streptozocin-induced diabetic mouse model (12). The expression of clock genes was altered in retina of diabetic mice (32), and the loss of core clock gene was related to the phenotype of DR. For instance, *Per2* mutant mice showed DR-like retinal vascular, including increased retinal vascular permeability and avascular capillaries (13). Interestingly, deletion of another core clock gene *Bmal1*, reduced neovascularization and vascular leakage, indicating *Bmal1* could drive neovascularization (33). In a summary, circadian rhythm disruption and altered expression of clock genes play crucial roles in the development of DR. The development of wearable device Actigraphy enables individuals to conduct 24-hour sleep and activity monitoring. The introduction of the RAR, generated from Actigraphy data, provides a robust quantification of daily rhythm. Recently, researchers have uncovered the association between RAR and various diseases, such as Parkinson’s disease, non-alcoholic fatty liver disease (34, 35). Additionally, it was found that blunted RAR is strongly correlated with biological aging and may negatively affect well-being and longevity (36). However, there are limited studies on relationship between RAR and DR.

Recently, Mayuko et al. examined RAR in relation to common diabetic complications, including DR (37). The findings of our study share similarities with those of Mayuko et al., but also exhibit differences. They stated that the presence of DR was associated with higher IV and lower daytime activity, consistent with our findings. However, Mayuko et al. found that RA and L5 were also significantly associated with DR, which was not observed in our study. The differences could be explained by a few reasons. Firstly, considering the complex survey design, sample weights for each individual was calculated when conducting analysis in our study, whereas Mayuko et al. had a limited sample size. Secondly, Mayuko et al. further graded DR into simple diabetic retinopathy and proliferative diabetic retinopathy, whereas we only considered the presence of DR without considering its severity.

M10, representing the mean activity level during the most active 10-hour period within 24-hour cycle, which is used to analyze activity patterns. In this study, M10 was found to be negatively associated with the risk of DR. It is well known that environmental and lifestyle modifications can alter circadian rhythm (38). Research in animals has shown that db/db mice, a well-acknowledged diabetic mouse model, showed sporadic wheel-running activity with no consistent pattern (39). There is substantial evidence supporting the insight that increasing physical activity alleviates the progression of DR (40). Several prospective cohort studies have reported that higher physical activity is independently associated with a lower incidence of DR (41, 42). The mechanism is speculated to be that physical activity might improve glycemic control and 25-hydroxyvitamin D level (43, 44). It was reported that 25-hydroxyvitamin D could inhibit retinal inflammation and retinal neovascularization *in vivo* and *in vitro (* 45–47). Thus, we speculate that subjects with high activity pattern (high M10 value) may have better glycemic control and higher level of 25-hydroxyvitamin D, thereby ameliorating DR-related pathogenesis.

Intradaily variability (IV), refers to fluctuation or change in an individual’s activity levels and rest periods within a single day. High IV indicates a less stable or more fragmented rest-activity pattern, with frequent transitions between activity and rest states. This instability generally results from irregular sleep patterns, inconsistent wake times or frequent napping. A case-control study compared sleep quality among diabetic patients with and without DR, and found that poor sleep, sleep latency and daytime dysfunction were more common in DR patients (48). A recent meta-analysis also reported poor sleep satisfaction was associated with a 2-fold risk of having DR (48). Sirimon et al. found DR patients were more likely to have insomnia and greater sleep variability (49). Melatonin, the primary hormone involved in regulating the sleep-wake cycles, is known that melatonin deficiency is responsible for sleep disorders (50). Melatonin exhibits multiple protective effects that could benefit DR patients, including anti-oxidative, anti-inflammatory and anti-apoptotic properties (51). Accordingly, we speculate that some participants, due to a lack of melatonin, may lose its protective effect on the retina and become more prone to developing DR. Additionally, melatonin-deficient participants are more likely to experience sleep disorders or fragmented rest-activity patterns, as evidenced by elevated IV value in RAR.

The main strength of this study is its nationally representative and diverse population-based cohort, which enhances the generalizability of the findings. However, there are several limitations to consider when interpreting the results. Firstly, due of the cross-sectional study design, causality between RAR metrics and DR cannot be established. Given that advanced stages of DR can cause severe vision loss and dysfunction of ipRGCs, leading to misalignment of circadian photoentrainment, it is possible that blunted RAR is a result of DR. Secondly, the diagnosis of DR was obtained from the self-reports in the medical conditions questionnaire, which could lead to diagnostic omissions. Additionally, in this study, only the presence of DR was available for analysis without considering the severity of DR. Future research should incorporate fundus photo and optical coherence tomography images for more accurate DR diagnosis and grading.

## 5 Conclusions

We demonstrated that altered RAR parameters are associated with the onset of DR. Notably, the significant associations were observed for IV, reflecting the fragmentation of rhythm, and for M10, reflecting the daytime activity level. These findings indicate that the fragmented rhythm and lower peak activity may increase the risk of DR. Further interventional research aimed at improving circadian rhythm through lifestyle, behavioral and environmental modification should be conducted to elucidate the casual links between circadian disruption and DR.

## Data Availability

The original contributions presented in the study are included in the article/in **Supplementary Material**. Further inquiries can be directed to the corresponding author.
