# Supplementary material for: Association between rest-activity rhythm and diabetic retinopathy among US middle-age and older diabetic adults
Source: Front Endocrinol (Lausanne). 2024 Sep 16;15:1440223. doi: 10.3389/fendo.2024.1440223 (PMC11439719; doi:10.3389/fendo.2024.1440223)
Supplement: Supplementary file 1 [file Table1.docx]

**Supplementary Table 1. Stratified analysis of M10 on the risk of DR.**

|  |  | OR (95% CI) | | | | |  |
| --- | --- | --- | --- | --- | --- | --- | --- |
| Subgroup | No. of participants (%) | Q1 | Q2 | Q3 | Q4 | Q5 | p for interaction |
| Age |  |  |  |  |  |  | 0.655 |
| 40-60 | 373(40.49) | ref | 0.498(0.187,1.331) | 0.559(0.167,1.867) | 0.286(0.090,0.904) | 0.524(0.126,2.182) |  |
| >=60 | 723(59.51) | ref | 0.761(0.309, 1.875) | 0.371(0.154, 0.897) | 0.422(0.165, 1.081) | 0.443(0.158, 1.239) |  |
| Sex |  |  |  |  |  |  | 0.439 |
| Female | 548(50.43) | ref | 1.030(0.327,3.245) | 0.458(0.176,1.191) | 0.284(0.108,0.751) | 0.444(0.128,1.541) |  |
| Male | 548(49.57) | ref | 0.500(0.235,1.063) | 0.390(0.134,1.135) | 0.395(0.150,1.036) | 0.640(0.226,1.815) |  |
| Race |  |  |  |  |  |  | 0.787 |
| Non-Hispanic White | 387(63.27) | ref | 0.507(0.176,1.463) | 0.358(0.136,0.942) | 0.231(0.067,0.790) | 0.510(0.109,2.384) |  |
| Other | 709(36.73) | ref | 1.016(0.480,2.151) | 0.524(0.240,1.145) | 0.514(0.235,1.124) | 0.707(0.332,1.505) |  |
| PIR |  |  |  |  |  |  | 0.336 |
| <1.3 | 447(28.70) | ref | 0.401(0.176,0.913) | 0.369(0.156,0.875) | 0.213(0.075,0.605) | 0.242(0.098,0.598) |  |
| 1.3-3.5 | 415(39.69) | ref | 1.167(0.339,4.020) | 0.427(0.128,1.426) | 0.587(0.198,1.735) | 0.696(0.201,2.412) |  |
| >3.5 | 234(31.61) | ref | 1.080(0.253, 4.619) | 1.060(0.191, 5.879) | 0.699(0.140, 3.505) | 1.964(0.287,13.451) |  |
| Education level |  |  |  |  |  |  | 0.149 |
| < High school | 370(23.83) | ref | 0.529(0.180,1.551) | 0.090(0.021,0.386) | 0.486(0.149,1.582) | 0.304(0.097,0.953) |  |
| High School Grad/GED or Equivalent | 256(25.51) | ref | 0.694(0.129,3.725) | 0.731(0.167,3.207) | 0.180(0.031,1.053) | 0.384(0.056,2.650) |  |
| > High school | 470(50.65) | ref | 0.990(0.429, 2.287) | 0.702(0.269, 1.833) | 0.404(0.161, 1.012) | 1.176(0.375, 3.692) |  |
| Martial status |  |  |  |  |  |  | 0.346 |
| Married or living with a partner | 598(60.35) | ref | 0.906(0.364,2.254) | 0.417(0.137,1.273) | 0.434(0.154,1.220) | 0.805(0.194,3.345) |  |
| Never married | 96(7.96) | ref | 0.280(0.038, 2.054) | 0.404(0.042, 3.872) | 0.127(0.005, 3.342) | 0.036(0.003, 0.464) |  |
| Widowed,divorced,seperated | 402(31.69) | ref | 0.539(0.194,1.503) | 0.405(0.152,1.080) | 0.207(0.074,0.574) | 0.435(0.147,1.288) |  |

Abbreviation: OR, odds ratios. CI, confidence interval. PIR, poverty income ratio. RAR, rest-activity rhythm. RA, relative amplitude. IS, interdaily stability. IV, intradailty variability. M10, most active 10-hour period. L5, least active 5-hour period. Ref, reference.
