# Supplementary material for: Association between rest-activity rhythm and diabetic retinopathy among US middle-age and older diabetic adults
Source: Front Endocrinol (Lausanne). 2024 Sep 16;15:1440223. doi: 10.3389/fendo.2024.1440223 (PMC11439719; doi:10.3389/fendo.2024.1440223)
Supplement: Supplementary file 2 [file Table2.docx]

**Supplementary Table 2. Stratified analysis of IV on the risk of DR.**

|  |  | OR (95% CI) | | | | |  |
| --- | --- | --- | --- | --- | --- | --- | --- |
| Subgroup | No. of participants (%) | Q1 | Q2 | Q3 | Q4 | Q5 | p for interaction |
| Age |  |  |  |  |  |  | 0.705 |
| 40-60 | 373(40.49) | ref | 1.165(0.357,3.799) | 1.130(0.428,2.982) | 0.761(0.242,2.400) | 2.288(0.598,8.763) |  |
| >=60 | 723(59.51) | ref | 2.380(1.024, 5.531) | 1.241(0.467, 3.301) | 1.972(0.566, 6.879) | 2.606(1.196, 5.682) |  |
| Sex |  |  |  |  |  |  | 0.775 |
| Female | 548(50.43) | ref | 2.302(0.910,5.820) | 1.013(0.283,3.621) | 1.453(0.426,4.955) | 2.378(0.689,8.210) |  |
| Male | 548(49.57) | ref | 1.225(0.427,3.511) | 1.343(0.658,2.743) | 1.237(0.452,3.388) | 2.048(1.130,3.710) |  |
| Race |  |  |  |  |  |  | 0.422 |
| Non-Hispanic White | 387(63.27) | ref | 2.885(0.713,11.671) | 1.964(0.569, 6.775) | 2.033(0.429, 9.626) | 2.917(0.825,10.314) |  |
| other | 709(36.73) | ref | 1.053(0.525,2.114) | 0.573(0.307,1.069) | 0.919(0.486,1.739) | 1.770(0.979,3.200) |  |
| PIR |  |  |  |  |  |  | 0.093 |
| <1.3 | 447(28.70) | ref | 2.353(0.778, 7.116) | 1.746(0.574, 5.313) | 2.071(0.732, 5.855) | 7.076(2.985,16.776) |  |
| 1.3-3.5 | 415(39.69) | ref | 1.593(0.687,3.692) | 0.482(0.169,1.379) | 1.049(0.243,4.520) | 1.306(0.450,3.795) |  |
| >3.5 | 234(31.61) | ref | 1.203(0.215, 6.724) | 1.843(0.491, 6.922) | 1.116(0.330, 3.781) | 0.897(0.216, 3.727) |  |
| Education level |  |  |  |  |  |  | 0.014* |
| < High school | 370(23.83) | ref | 2.776(1.255, 6.141) | 0.952(0.324, 2.800) | 1.480(0.354, 6.189) | 4.921(1.750,13.841) |  |
| High School Grad/GED or Equivalent | 256(25.51) | ref | 12.350(2.166,70.418) | 7.059(1.403,35.512) | 6.880(1.130,41.906) | 9.809(2.038,47.205) |  |
| > High school | 470(50.65) | ref | 0.385(0.093,1.586) | 0.460(0.131,1.618) | 0.649(0.227,1.852) | 0.677(0.208,2.208) |  |
| Martial status |  |  |  |  |  |  | 0.2 |
| Married or living with a partner | 598(60.35) | ref | 0.960(0.377,2.443) | 0.951(0.448,2.017) | 0.900(0.315,2.573) | 1.146(0.463,2.834) |  |
| Never married | 96( 7.96) | ref | 6.286(0.398, 99.267) | 0.636(0.035, 11.607) | 1.264(0.047, 34.291) | 14.017(0.585,335.927) |  |
| Widowed,divorced,seperated | 402(31.69) | ref | 3.975(1.485,10.644) | 1.332(0.462, 3.839) | 2.165(0.971, 4.829) | 4.137(1.600,10.698) |  |

Abbreviation: OR, odds ratios. CI, confidence interval. PIR, poverty income ratio. RAR, rest-activity rhythm. RA, relative amplitude. IS, interdaily stability. IV, intradailty variability. M10, most active 10-hour period. L5, least active 5-hour period. Ref, reference.
