# Supplementary material for: Association between rest-activity rhythm and diabetic retinopathy among US middle-age and older diabetic adults
Source: Front Endocrinol (Lausanne). 2024 Sep 16;15:1440223. doi: 10.3389/fendo.2024.1440223 (PMC11439719; doi:10.3389/fendo.2024.1440223)
Supplement: Supplementary file 3 [file Table3.docx]

Supplementary Table 3. Association between RAR metrics and DR (N=938).

| 24-h Rest-activity rhythm variables | Model 1 | | Model 2 | | Model 3 | |
| --- | --- | --- | --- | --- | --- | --- |
|  | OR (95% CI) | p | OR (95% CI) | p | OR (95% CI) | p |
| IS | 0.156(0.015,1.627) | 0.116 | 0.162(0.016,1.603) | 0.114 | 0.161(0.016,1.650) | 0.118 |
| IV | 3.467(0.899,13.366) | 0.070 | 4.441(1.253,15.739) | 0.023* | 4.441(1.238,15.934) | 0.024* |
| L5 | 0.995(0.792,1.252) | 0.968 | 0.974(0.771,1.229) | 0.814 | 0.974(0.772,1.228) | 0.813 |
| M10 | 0.895(0.798,1.004) | 0.057 | 0.874(0.781,0.977) | 0.020* | 0.874(0.780,0.978) | 0.022* |
| RA | 0.252(0.040,1.578) | 0.136 | 0.273(0.045,1.661) | 0.151 | 0.272(0.044,1.675) | 0.152 |

Abbreviation: OR, odds ratios. CI, confidence interval. RAR, rest-activity rhythm. RA, relative amplitude. IS, interdaily stability. IV, intradailty variability. M10, most active 10-hour period. L5, least active 5-hour period. Ref, reference.
