# Supplementary material for: Association between rest-activity rhythm and diabetic retinopathy among US middle-age and older diabetic adults
Source: Front Endocrinol (Lausanne). 2024 Sep 16;15:1440223. doi: 10.3389/fendo.2024.1440223 (PMC11439719; doi:10.3389/fendo.2024.1440223)
Supplement: Supplementary file 4 [file Table4.docx]

**Supplementary Table 4. Baseline characteristics of participants stratified by Clusters based on IV and M10.**

| Characteristic | Cluster A  (N=230) | Cluster B  (N=519) | Cluster C  (N=347) | p |
| --- | --- | --- | --- | --- |
| Age, n (%) |  |  |  | < 0.001 |
| 40-60 | 110(55.8107) | 199(41.5787) | 64(28.4306) |  |
| ≥60 | 120(44.1893) | 320(58.4213) | 283(71.5694) |  |
| Sex, n (%) |  |  |  | 0.0084 |
| Female | 138(59.7522) | 272(52.7059) | 138(40.9853) |  |
| Male | 92(40.2478) | 247(47.2941) | 209(59.0147) |  |
| Race, n (%) |  |  |  | < 0.001 |
| Non-Hispanic White | 52(46.2367) | 197(66.5075) | 138(67.3823) |  |
| other | 178(53.7633) | 322(33.4925) | 209(32.6177) |  |
| Education level, n (%) |  |  |  | 0.0121 |
| < High school | 99(36.4272) | 143(20.3295) | 128(23.6959) |  |
| High School Grad/GED or Equivalent | 54(22.7043) | 125(27.0209) | 77(25.2854) |  |
| > High school | 77(40.8685) | 251(52.6495) | 142(51.0187) |  |
| Martial status, n (%) |  |  |  | 0.5502 |
| Married or living with a partner | 126(55.3411) | 298(62.9657) | 174(57.5661) |  |
| Never married | 23(9.9239) | 41(7.3236) | 32(8.3191) |  |
| Widowed,divorced,seperated | 81(34.7350) | 180(29.7106) | 141(34.1148) |  |
| PIR, n (%) |  |  |  | < 0.001 |
| <1.3 | 113(42.6199) | 175(21.5802) | 159(34.4467) |  |
| 1.3-3.5 | 80(37.2098) | 213(42.3528) | 122(36.0262) |  |
| >3.5 | 37(20.1702) | 131(36.0669) | 66(29.5271) |  |
| Smoke, n (%) |  |  |  | 0.1271 |
| never | 140(56.0947) | 252(45.3959) | 158(50.5075) |  |
| former | 61(30.5869) | 184(38.4544) | 148(39.5635) |  |
| now | 29(13.3184) | 83(16.1497) | 41( 9.9290) |  |
| Hypertension, n (%) |  |  |  | < 0.0001 |
| No | 88(41.3658) | 117(22.2615) | 52(14.4965) |  |
| Yes | 142(58.6342) | 402(77.7385) | 295(85.5035) |  |
| Hyperlipidemia, n (%) |  |  |  | 0.38 |
| No | 36(13.4842) | 67( 9.4485) | 35( 8.5651) |  |
| Yes | 194(86.5158) | 452(90.5515) | 312(91.4349) |  |
| Sleep problem, n (%) |  |  |  | 0.4474 |
| No | 155(64.8361) | 322(59.5588) | 215(55.6817) |  |
| Yes | 75(35.1639) | 197(40.4412) | 132(44.3183) |  |
| DR, n (%) |  |  |  | 0.0066 |
| No | 188(82.7021) | 433(87.2317) | 250(75.5056) |  |
| Yes | 42(17.2979) | 86(12.7683) | 97(24.4944) |  |
| IS, mean±SD | 0.4242±0.0063 | 0.3759±0.0050 | 0.3248±0.0070 | < 0.0001 |
| IV, mean±SD | 0.4819±0.0102 | 0.6198±0.0095 | 0.7920±0.0151 | < 0.0001 |
| RA, mean±SD | 0.8569±0.0071 | 0.8027±0.0071 | 0.7408±0.0132 | < 0.0001 |
| L5, mean±SD | 1.1130±0.0623 | 1.1387±0.0539 | 0.9573±0.0585 | 0.0849 |
| M10, mean±SD | 14.0609±0.1029 | 9.9302±0.0745 | 6.3325±0.0692 | < 0.0001 |

Abbreviation: PIR, poverty income ratio. IS, interdaily stability. IV, intradaily variability. M10, most active 10-hour period. L5, least active 5-hour period. RA, relative amplitude.
